# Effects of running fatigue on lower extremity symmetry among amateur runners: From a biomechanical perspective

**DOI:** 10.3389/fphys.2022.899818

**Published:** 2022-09-02

**Authors:** Zixiang Gao, Gusztáv Fekete, Julien S. Baker, Minjun Liang, Rongrong Xuan, Yaodong Gu

**Affiliations:** ^1^ Faculty of Sports Science, Ningbo University, Ningbo, China; ^2^ Faculty of Engineering, University of Pannonia, Veszprém, Hungary; ^3^ Savaria Institute of Technology, Eötvös Loránd University, Szombathely, Hungary; ^4^ Department of Sport and Physical Education, Hong Kong Baptist University, Hong Kong, Hong Kong SAR, China; ^5^ Department of Physical and Health Education, Udon Thani Rajabhat University, Udon Thani, Thailand; ^6^ The Affiliated Hospital of Medical School of Ningbo University, Ningbo University, Ningbo, China

**Keywords:** symmetry angle, running-induced fatigue, dominant limb, statistical parametric mapping, bilateral

## Abstract

The objective of this study was to examine the effects of running fatigue on the symmetry of lower limb biomechanical parameters in eighteen male amateur runners. The marker trajectories and ground reaction forces were collected before and after the running-induced fatigue protocol. Symmetry angles (SA) were used to quantify the symmetry of each parameter. Normality tests and Paired sample T-tests were carried out to detect bilateral lower limb differences and SA of parameters between pre- and post-fatigue. One-dimensional statistical parameter mapping (SPM_1d) was used to compare parameters with the characteristic of one-dimensional time series data of lower limbs. After fatigue, the SA of knee extension angles, knee abduction moment, and hip joint flexion moment increased by 17%, 10%, and 11% respectively. In comparison, the flexion angle of the knee joint decreased by 5%. The symmetry of internal rotation of ankle, knee and hip joints increased after fatigued, while the SA of external rotation of the three joints decreased significantly. These findings provide preliminary evidence that fatigue changes lower limb symmetry in running gait and may have implications for understanding running-related injuries and performance.

## Introduction

It is estimated that more than 35 million Americans participate in long-distance running as part of their daily physical activity (Wouters et al., 2012). Previous studies have shown that individuals with a long distance running habit can reduce the risk of cardiovascular-related death by 45%–70% (Lee et al., 2014) and cancer-related death by 30–50% (Chakravarty et al., 2008). However, fatigue from long distance running is also associated with a higher rate of injuries (Van Gent et al., 2007). Hulme et al. (Hulme et al., 2017) reported that 2.5–33.0 running-related injuries occurred per 1,000 h of running, and more than 79.3% of injuries occurred in knee joints. In addition, changes in muscle strength, cognitive function, and proprioception can be caused by fatigue (Abd-Elfattah et al., 2015), besides, stress, strain, shear force, and impact force on the lower limb joints also increase during fatigue (Dierks et al., 2010). Asymmetry between limbs refers to the phenomenon that one limb difference of function, physical strength, and other parameters relative to the other limb (Sadeghi et al., 2000). Previous study has reported that the healthy individuals who exhibit asymmetries (symmetry score>15%) have mare relationship with rise in injury incidence rate compared to individuals who symmetry score below 15% (Barber et al., 1990). In addition, a recent systematic review on prospective evidence for running related injury found only limited evidence for increased asymmetry in ground contact time and decreased asymmetry in vertical impact peak as being related to running injury (Ceyssens et al., 2019). Consequently, the presence of bilateral lower limbs asymmetry may be one of the potential causes of injure. Especially when one side of the limb load is more than the other side, suggesting that the unilateral injures may occur (Furlong and Egginton, 2018). For example, healthy recreational runners have significantly higher Achilles tendon loads in the dominant lower limbs than in the non-dominant lower limbs (Furlong and Egginton, 2018). Part of the reason for gait asymmetry is the difference in functional tasks, which is mainly explained by the contribution of control and propulsion degree (Sadeghi et al., 2000). Moreover, Seeley et al. (Seeley et al., 2008) found that impulses from dominant limbs were significantly larger than those from non-dominant limbs during the push-off phase during fast walking, suggesting that the dominant limb contribute more to gait propulsion. Likewise, previous studies also have reported that the non-dominant foot showed more stable Foot Balance Index Range (FBIR) during running gait (Gao et al., 2020a).

Furthermore, asymmetries in running can directly affect the quality of running, Beck et al. (Beck et al., 2018) showed that running increased the level of foot contact time asymmetry by 10% and its metabolic cost increased by 7.8%. In addition, a 10% increase in the asymmetry of mean ground reaction forces resulted in a 3.5% increase in metabolic costs. Exercise-induced fatigue may cause or exacerbate pre-existing limb asymmetries (Bell et al., 2016). This change may be a deterioration in movement patterns due to fatigue resulting in poor neuromuscular control, proprioception, postural control, or motor coordination (Smeets et al., 2019). Likewise, Gao et al. (Gao et al., 2020a) reported that a Running-Induced Fatigue Protocol caused knee flexion angle, hip flexion angle, hip extension angle, and the hip flexion moment to be more asymmetrical. However, the biomechanical changes of human movement usually occur in three anatomical planes (Cen et al., 2021). Therefore, the effect of fatigue on the symmetry of coronal and horizontal biomechanical parameters is less known.

Symmetry changes caused by fatigue may be one of the factors leading to running-related injuries and an economic decline in running performance. Therefore, the aim of this study was to investigate if there is asymmetry in the kinematic and dynamic parameters of lower extremity joints before and after fatigue. In addition, we also examined whether fatigue could impact negatively on asymmetry. Therefore, three hypotheses were proposed in this study: 1) In addition to sagittal plane, biomechanical asymmetry also exists in coronal plane and horizontal plane before running fatigue protocol. 2) The asymmetric parameters change after the implementation of running fatigue protocol. 3) The symmetry of kinematic and kinetics parameters decreases significantly after the Running-induced Fatigue Protocol.

## Methods

### Participants

Eighteen healthy male amateur runners were recruited for this study (Years: 22.72 ± 1.40-years, Height: 174.72 ± 6.75 cm, Mass: 69.72 ± 7.26 kg, BMI: 22.67 ± 2.16 kg/m^2^). All subjects’ dominant limb was the right leg, defined as the preferred leg when kicking a ball. Amateur runners were defined as running at least 2–3 times a week for less than 45 min or running less than 10 km. In the previous 6 months, subjects with trunk, pelvis, lower limb injuries and a score of 72 or above on the 5-point Lower Extremity Functional Scale [LEFS, the scale consisted of 20 items, which form unable to perform activity (0-score) to no difficulty (5-score)] were excluded from the study (Binkley et al., 1999). The study was approved by the ethics committee from the Research Institute in the University.

### Experimental protocol

**FIGURE 1:**
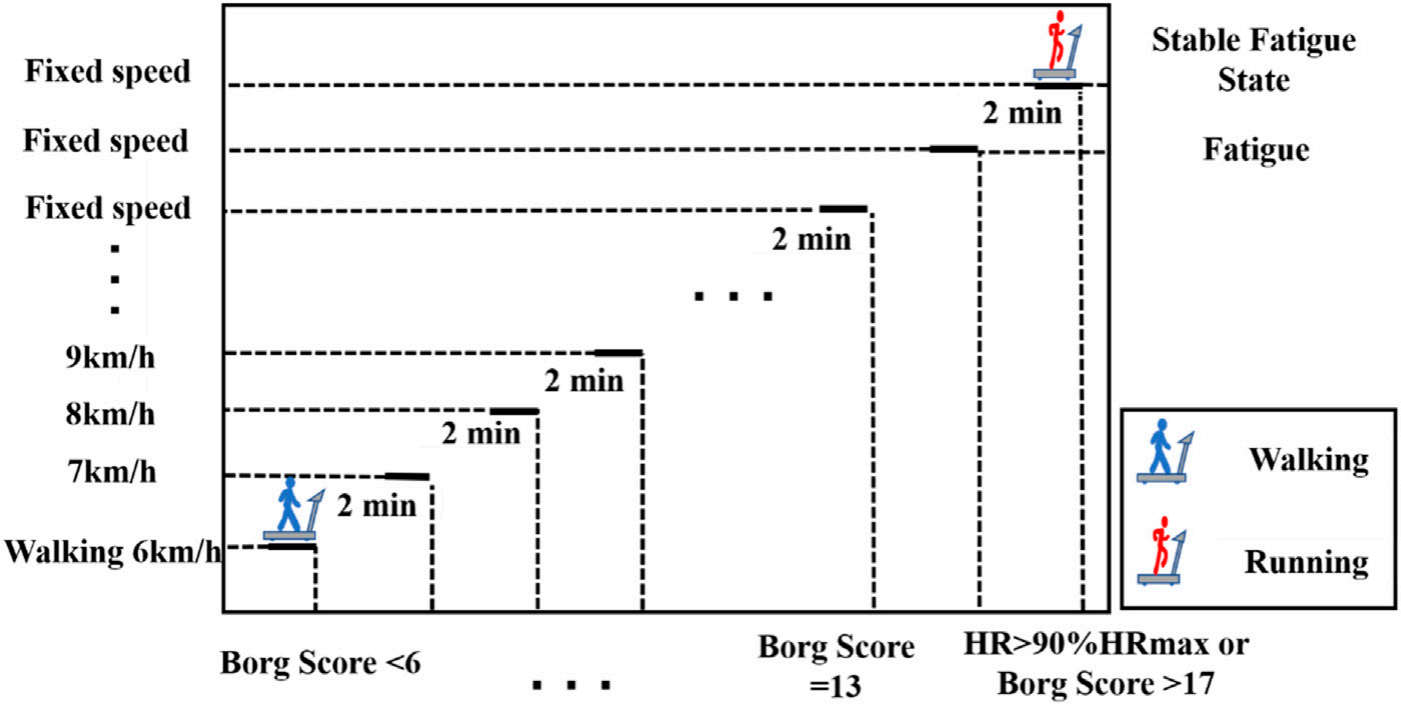
Execution method of running-induced fatigue experiment.

**FIGURE 2:**
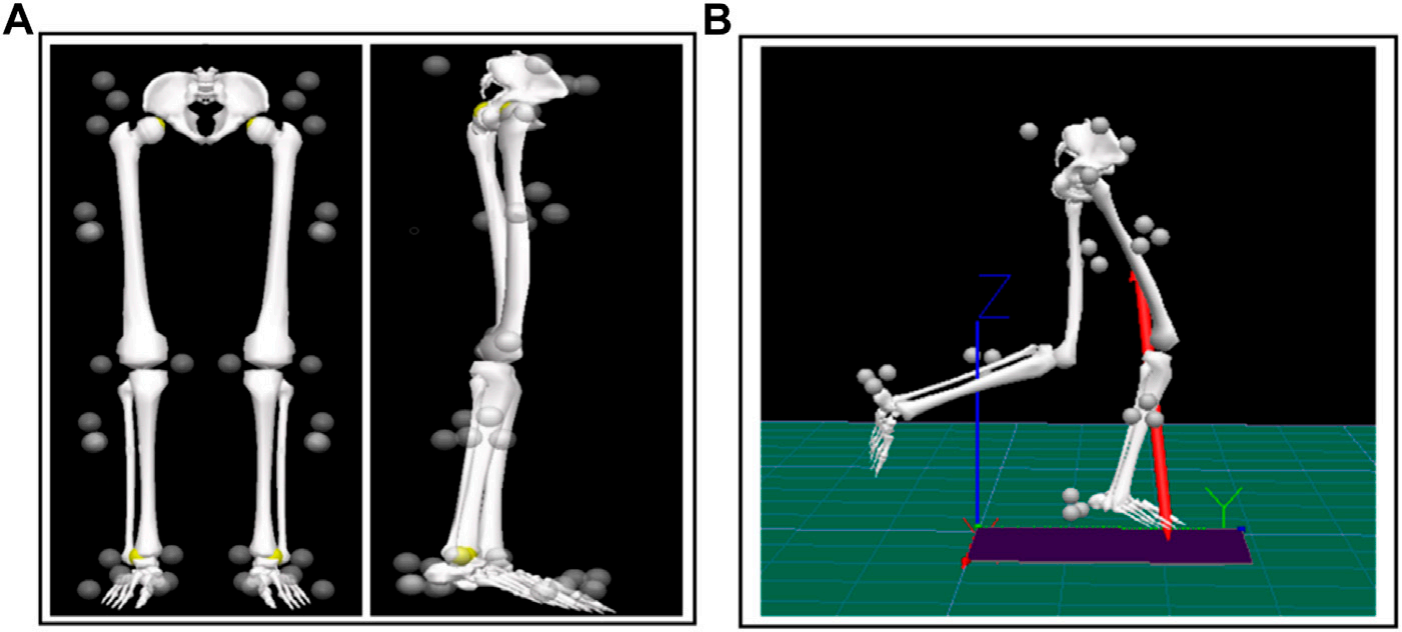
The placement of reflective markers as well as static and dynamic modeling in V3D. **(A)** The placement of reflective markers and static modelling; **(B)** Establishment of dynamic running model in V3D.

Subjects were required to be familiar with the experimental environment and procedures prior to the experiment. Prior to the commencement of data collection all subjects participated in a warmup comprising of a 5-minute jog on the treadmill (Satun h/p/cosmos, Germany). All subjects wore standard running shoes provided by the laboratory. Subsequently, the Over-ground running test before and after the Running-induced Fatigue Protocol were successively implemented. A Vicon eight-camera motion capture system (Vicon Metrics Ltd., Oxford, United Kingdom) and a Kistler Force plate (Kistler, Winterthur, Switzerland) were used to collect the marker trajectories and ground reaction force signals. The frequencies were 200 Hz and 1000 Hz, respectively. Twenty-one reflective markers and six reflective marker clusters were attached to the anatomy of the pelvis, left and right thighs, calves, and feet to define the hip, knee, and ankle joints for kinematic data collection (Figure 2) (Hannigan and Pollard, 2020). Participants were asked to run through the 10-m signal acquisition area at a comfortable speed. Four successful left and right leg tests were collected before and after the Running-induced Fatigue Protocol. According to the Running-induced Fatigue Protocol of Koblbauer et al. (Koblbauer et al., 2014), individual subject per-minute fatigue level, and pre-minute was digitally monitored using heart rate telemetry and the 15-point Borg scale (Figure 1). Subjects were limited to performing the post-fatigue ground running test within 5 min of the end of the Running-induced Fatigue Protocol (Mei et al., 2019). The specific execution details of running-induced Fatigue have been previously described (Koblbauer et al., 2014; Gao et al., 2020a).

### Data processing

Visual 3D (c-motion Inc., Germantown, MD, United States) human motion analysis software was used to establish the statical and dynamic running model (Figure 2) (Hannigan and Pollard, 2020). The model was created using recorded static marker positions and anthropometric parameters (height and mass) of the participants under test. Inverse kinematics algorithm was used to calculate joint angles in 3- dimensions visual environment. The joint moment is obtained by inputting the calculated joint Angle and the collected ground reaction force and performing the inverse dynamics algorithm. In order to avoid experimental errors caused by individual differences, the joint moment data were standardized using individual subject weight.

The Symmetry Angle (SA) is commonly used to evaluate the relationship between the left and right discrete values (Zifchock et al., 2008). The principle is to use the angle formed by the vector plotted by the right value and the left value in the coordinate system to evaluate symmetry:

$$\mathrm{SA}\left(\%\right)=\frac{\left(45{}^{\circ}-\arctan\left(\frac{X_{\mathrm{left}}}{X_{\mathrm{right}}}\right)\right)}{90{}^{\circ}}\times 100\%$$ (1)

Where the Xleft and Xright represents the left and right limb values respectively. SA with a value of 0% represents perfect symmetry, and 100% represents two values equal in magnitude and opposite in direction (complete asymmetry). If [45°-arctan (Xleft/Xright)] > 90°, the following equation should be substituted:

$$\mathrm{SA}\left(\%\right)=\frac{\left(45{}^{\circ}-\arctan\left(\frac{X_{\mathrm{left}}}{X_{\mathrm{right}}}\right)\right)-180{}^{\circ}}{90{}^{\circ}}\times 100\%$$ (2)

Stiffness is associated with injury and performance in neuromuscular controlled movements (Butler et al., 2003). Joint stiffness has been associated with overuse injury in previous studies because of compliant joint contributes more to attenuation of joint load than a stiffer joint (Hamill et al., 2009). Therefore, we use joint stiffness to quantify the interaction between ROM and the joint moment.

Kjoint=ΔM/ROM (3)

ΔM is defined as the change of moment of the ankle, knee, and hip joints during the stance phase. ROM is defined as the range of motion of the joint during the stance phase (Brughelli and Cronin, 2008).

### Statistical analysis

The Shapiro-Wilks test was used to verify the normality of the data using SPSS (Version 19; SPSS, Inc., Chicago, IL, United States). Paired sample T-tests were used to examine peak joint moment, joint stiffness. Paired sample T-tests also were used to examine the SA for peak joint angle, moment and stiffness in the bilateral lower extremities before and after fatigue. The open source algorithm of one-dimensional Statistical Parameter Mapping (SPM_1d, paired sample *t*-test algorithm package) was used in MATLAB R2018a software to perform the statistical tests on the joint continuity angle (Gao et al., 2020b). Significance levels were set at 0.05.

## Results

### Kinematics parameters

**FIGURE 3:**
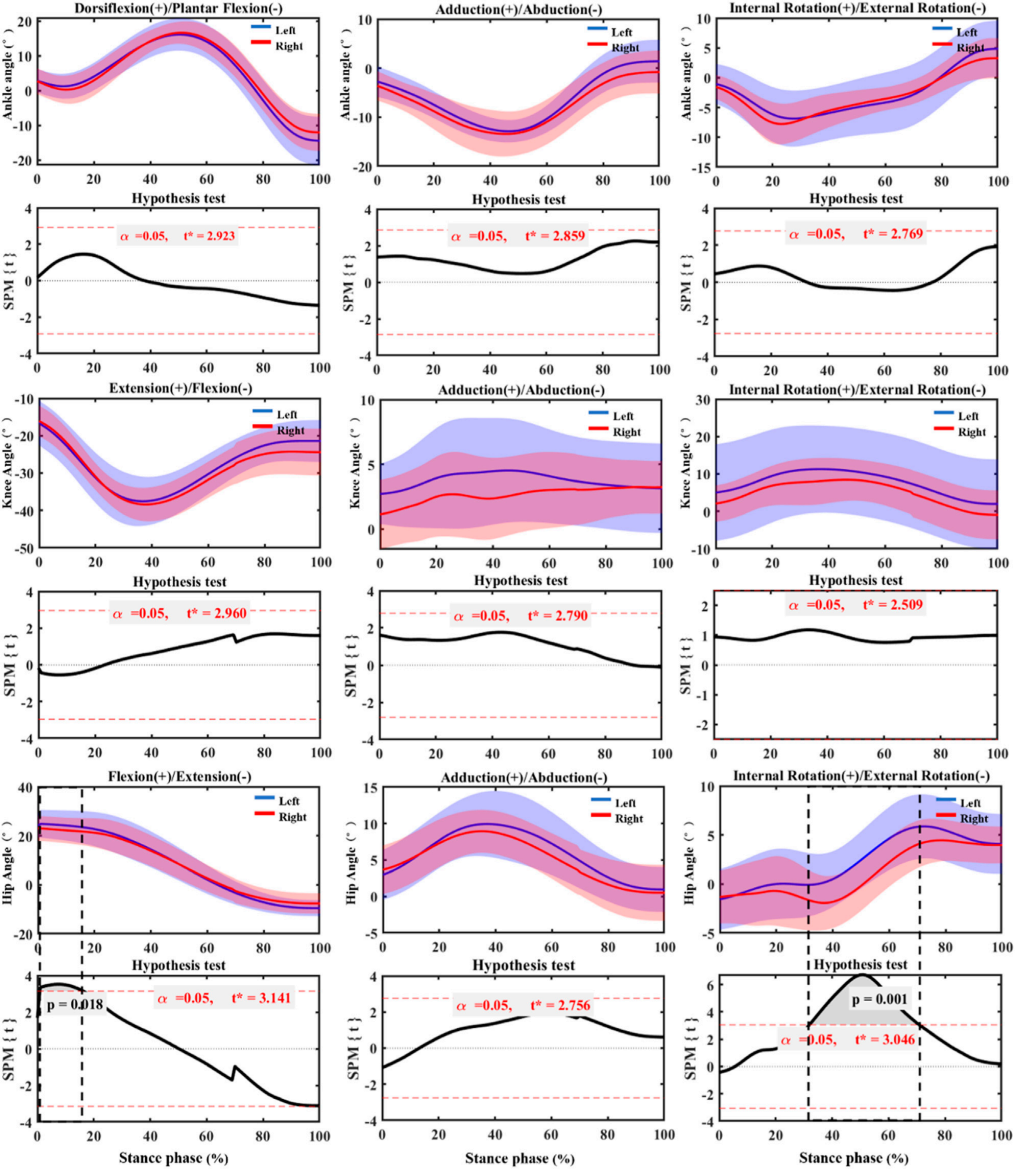
SPM_1d test of joint angle changes of bilateral lower extremities before fatigue during stance phase. Left: Left lower limb (non-dominant). Right: Right (dominant) lower limb.

According to the SPM_1d test results of pre-fatigue in Figure 3. The left hip joint showed significant flexion (*p* = 0.018), which was mainly manifested in the foot initial contact stage. Similarly, the external rotation of the left hip joint was significantly higher than the other side during the 32–72% of stance phase (*p* = 0.001).

**FIGURE 4:**
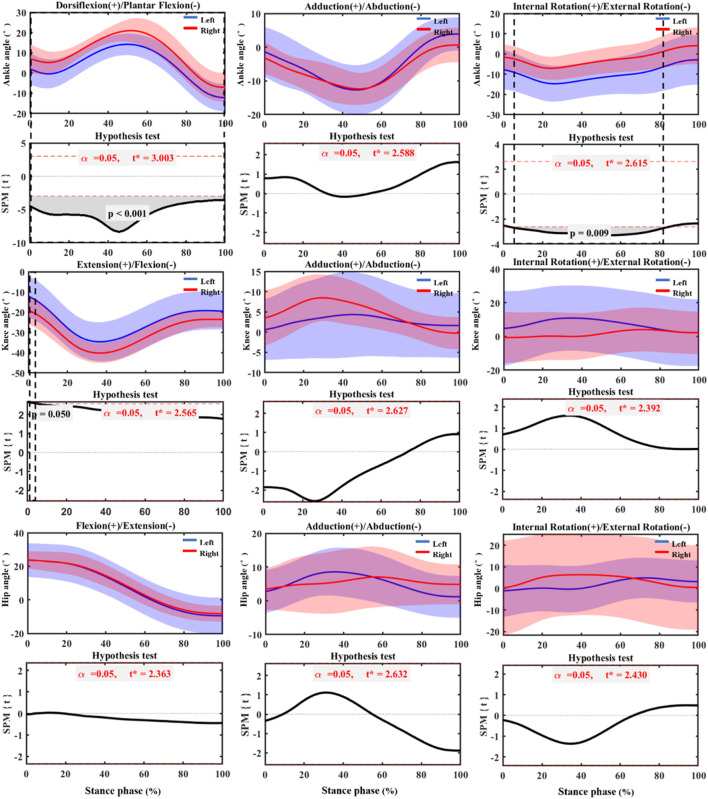
SPM_1d test of joint angle changes of bilateral lower limbs post fatigue during stance phase. Left: Left lower limb (non-dominant). Right: Right (dominant) lower limb.

According to the SPM_1d test results of post-fatigue in Figure 4, the right ankle showed very significant dorsiflexion during the entire stance phase compared with the left ankle (*p* < 0.001). In addition, the right ankle showed very significant internal rotation from the initial contact to the toe off stage (*p* = 0.009). Compared with the left knee, the right knee showed significant flexion immediately initial contact stage (*p* = 0.05).

### Kinetics parameters

The comparison of joint kinetics parameters of bilateral ankle, knee and hip joints as shown in Table 1. Before the running-induced fatigue test, the left ankle joint presented a very significant adduction moment compared with the other side (*p* = 0.005; ES = 0.40). In contrast, the significant abduction moment was observed in right ankle joint than the left side (*p* = 0.027; ES = 0.22). The left ankle showed greater joint stiffness in the coronal plane than the right side (*p* = 0.026; ES = 0.30). After the running-induced fatigue test, the peak adduction moment of the left ankle was observed significantly greater than right ankle (*p* = 0.013; ES = 0.42). In comparison, the peak abduction moment of the right ankle was greater (*p* = 0.003; ES = 0.40). The stiffness of the left ankle in the coronal plane was significantly higher than that of the right ankle (*p* = 0.030; ES = 0.29). In contrast, the stiffness of left ankle in the horizontal plane was lower than the other side (*p* = 0.039; ES = −0.17).

**TABLE 1:** Comparison of joint moment and joint stiffness of bilateral ankle, knee and hip joints before and after fatigue.

	Pre-fatigue mean (±SD)				Post-fatigue mean (±SD)			
	Left	Right	Sig.	ES (Cohen’s d)	Left	Right	Sig.	ES (Cohen’s d)
Ankle Joint Moment (Nm/kg)								
Dors (+)	0.15 (0.14)	0.11 (0.11)	0.126	0.16 (0.23)	0.10 (0.08)	0.11 (0.11)	0.656	−0.05 (0.10)
Flex (−)	−0.94 (0.91)	−0.62 (0.65)	0.101	−0.20 (−0.40)	−0.86 (0.81)	−1.07 (0.82)	0.377	0.13 (0.26)
Add (+)	0.24 (0.23)	0.08 (0.12)	0.005**	0.40 (0.87)	0.36 (0.41)	0.08 (0.10)	0.013**	0.42 (0.94)
Abd (−)	−0.01 (0.01)	−0.02 (0.03)	0.027*	0.22 (0.45)	−0.02 (0.02)	−0.06 (0.06)	0.003**	0.40 (0.89)
Intr (+)	0.02 (0.01)	0.02 (0.02)	0.934	0 (0)	0.02 (0.03)	0.06 (0.10)	0.051	−0.26 −0.54)
Extr (−)	−0.09 (0.08)	−0.06 (0.06)	0.138	−0.21	−0.05 (0.06)	−0.12 (0.14)	0.082	0.31 (0.65)
Ankle Joint stiffness (Nm/deg)								
Dors (+)/Flex (−)	0.04 (0.03)	0.03 (0.03)	0.177	0.16 (0.33)	0.04 (0.04)	0.04 (0.03)	0.643	0 (0)
Add (+)/Abd (−)	0.02 (0.02)	0.01 (0.01)	0.026*	0.30 (0.63)	0.02 (0.02)	0.01 (0.01)	0.030*	0.29 (0.60)
Intr (+)/Extr (−)	0.01 (0.01)	0.01 (0.01)	0.307	0 (0)	0.01 (0.01)	0.02 (0.04)	0.039*	−0.17 (−0.34)
Knee Joint Moment (Nm/kg)								
Ext (+)	0.50 (0.56)	0.31 (0.29)	0.037	0.21 (0.43)	0.58 (0.81)	0.74 (0.89)	0.131	−0.9 (−0.19)
Flex (−)	−1.05 (0.83)	−0.81 (0.68)	0.275	−0.15 (−0.32)	−0.93 (0.68)	−1.09 (0.61)	0.417	0.12 (0.25)
Add (+)	0.16 (0.11)	0.08 (0.07)	0.014*	0.40 (0.87)	0.26 (0.27)	0.10 (0.09)	0.007**	0.40 (0.80)
Abd (−)	−0.17 (0.18)	−0.16 (0.13)	0.764	−0.03 (−0.06)	−0.30 (0.42)	−0.26 (0.22)	0.771	−0.06 (−0.11)
Intr (+)	0.03 (0.03)	0.02 (0.02)	0.162	0.19 (0.39)	0.03 (0.04)	0.05 (0.06)	0.092	−0.19 (−0.39)
Extr (−)	−0.08 (0.07)	−0.04 (0.04)	0.050*	−0.80 (−2.63)	−0.07 (0.08)	−0.12 (0.10)	0.091	0.27 (0.55)
Knee Joint stiffness (Nm/deg)								
Ext (+)/Flex (−)	0.08 (0.07)	0.05 (0.04)	0.045*	0.25 (0.53)	0.07 (0.06)	0.09 (0.06)	0.242	−0.16 (−0.33)
Add (+)/Abd (−)	0.14 (0.14)	0.08 (0.07)	0.033*	0.26 (0.54)	0.31 (0.49)	0.04 (0.03)	0.027*	0.36 (0.78)
Intr (+)/Extr (−)	0.01 (0.01)	0.06 (0.01)	0.008**	−0.93 (−5.00)	0.01 (0.02)	0.02 (0.02)	0.080	−0.24 (−0.5)
Hip Joint Moment (Nm/kg)								
Flex (+)	0.75 (0.16)	0.74 (0.17)	0.726	0.03 (0.06)	0.71 (0.36)	0.66 (0.26)	0.377	0.08 (0.16)
Ext (−)	−2.14 (0.77)	−1.87 (0.63)	0.238	−0.19 (−0.38)	−1.8 (0.64)	−1.9 (0.49)	0.815	0.09 (0.18)
Add (+)	0.32 (0.16)	0.24 (0.17)	0.001**	0.24 (0.48)	0.40 (0.31)	0.36 (0.29)	0.560	0.07 (0.13)
Abd (−)	−0.49 (0.32)	−0.38 (0.30)	0.132	−0.17 (−0.35)	−0.55 (0.58)	−0.68 (0.28)	0.376	0.14 (0.29)
Intr (+)	0.11 (0.09)	0.10 (0.08)	0.491	0.06 (0.12)	0.15 (0.17)	0.17 (0.12)	0.611	−0.07 (−0.14)
Extr (−)	−0.14 (0.09)	−0.08 (0.08)	0.026*	−0.33 (−0.70)	−0.19 (0.20)	−0.08 (0.08)	0.015*	−0.334 (−0.72)
Hip Joint stiffness (Nm/deg)								
Flex (+)/Ext (−)	0.08 (0.02)	0.08 (0.02)	0.912	0 (0)	0.08 (0.03)	0.08 (0.02)	0.743	0 (0)
Add (+)/Abd (−)	0.09 (0.03)	0.07 (0.04)	0.124	0.27 (0.57)	0.11 (0.08)	0.21 (0.10)	0.009**	−0.48 (−1.01)
Intr (+)/Extr (−)	0.03 (0.02)	0.02 (0.02)	0.066	0.24 (0.5)	0.07 (0.08)	0.03 (0.02)	0.052	0.32 (0.69)

*means significance (*p* < 0.05), “**” means very significance (*p* < 0.01).

Left: Left lower limb (non-dominant). Right: Right (dominant) lower limb. Dors: Dorsiflexion, flex: Plantar flexion, Add: Adduction, Abd: Abduction, Intr: Internal rotation, Extr: External rotation. ES: effect Size.

Bold values means statistical significance.

Before the running-induced fatigue test, it can be seen form Table 1 that the peak adduction moment (*p* = 0.014; ES = 0.40) and the peak external rotation moment (*p* = 0.050; ES = −0.80) of the left knee joint was significantly greater than that of the right knee joint. The stiffness of the left knee joint was significantly greater than the right in the sagittal plane (*p* = 0.045; ES = 0.25) and coronal plane (*p* = 0.033; ES = 0.26), but it lower in the horizontal plane (*p* = 0.008; ES = −0.93). After the running-induced fatigue test, the peak adduction moment of the left knee joint was significantly greater than that of the right knee (*p* = 0.007; ES = 0.40). The stiffness of the left knee joint on the coronal plane was significantly higher than then right side (*p* = 0.027; ES = 0.36).

Before the running-induced fatigue test, the peak adduction moment (*p* = 0.001; ES = 0.24) and peak external rotation (*p* = 0.026; ES = −0.33) moment of the left hip joint showed significantly greater compared with the right side. After the running-induced fatigue test, the peak external rotation moment (*p* = 0.015; ES = −0.33) of the left hip joint was significantly more extensive than the other side and the stiffness of the right hip joint on the coronal plane was significantly higher than that on the left (*p* = 0.009; ES = −0.48).

### Symmetry parameter

As shown in Table 2, the SA of peak abduction angle of ankle joint before fatigue was significantly higher (19%) than that after fatigue (*p* = 0.000; ES = 0.55). The SA of the ankle peak internal rotation angle and peak external rotation moment (*p* = 0.006; ES = −0.39) was significantly increased by 51% and 13% after the running-induced fatigue test (*p* = 0.000; ES = −0.47). At the same time, the SA of the peak external rotation angle was significantly decreased by 10% after fatigued (*p* = 0.026; ES = 0.19).

**TABLE 2:** Changes of ankle joint angle, joint moment, range of motion and joint stiffness symmetry before and after fatigue.

Ankle	Symmetry angle (%)			
	Pre- mean (±SD)	Post- mean (±SD)	Sig.	ES (Cohen’s d)
Joint Angle				
Dors (+)	0.09 (0.08)	0.13 (0.07)	0.104	−0.26 (−0.53)
Flex (−)	0.18 (0.13)	0.28 (0.40)	0.377	−0.17 (0.34)
Add (+)	0.56 (0.51)	0.61 (0.42)	0.755	−0.05 (−0.11)
Abd (−)	0.21 (0.11)	0.09 (0.07)	0.000**	0.55 (1.30)
Intr (+)	0.43 (0.47)	0.94 (0.48)	0.000**	−0.47 (−1.07)
Extr (−)	0.30 (0.31)	0.20 (0.21)	0.026*	0.19 (0.38)
Moment				
Dors (+)	0.17 (0.15)	0.13 (0.07)	0.378	0.17 (0.34)
Flex (−)	0.22 (0.20)	0.25 (0.20)	0.585	−0.07 (−0.15)
Add (+)	0.32 (0.15)	0.14 (0.13)	0.103	0.54 (1.28)
Abd (−)	0.22 (0.15)	0.31 (0.12)	0.079	−0.31 (−0.66)
Intr (+)	0.23 (0.12)	0.30 (0.16)	0.160	−0.24 (−0.49)
Extr (−)	0.22 (0.16)	0.35 (0.15)	0.006**	−0.39 (−0.84)
Joint stiffness				
Dors (+)/Flex (−)	0.18 (0.16)	0.22 (0.20)	0.538	−0.11 (−0.22)
Add (+)/Abd (−)	0.28 (0.14)	0.29 (0.17)	0.809	−0.03 (−0.06)
Intr (+)/Extr (−)	0.24 (0.15)	0.32 (0.15)	0.076	−0.26 (−0.53)

*means significance (*p* < 0.05), “**” means very significance (*p* < 0.01).

Left: Left lower limb (non-dominant). Right: Right (dominant) lower limb. Dors: Dorsiflexion, flex: Plantar flexion, Add: Adduction, Abd: Abduction, Intr: Internal rotation, Extr: External rotation. ES: effect Size.

Bold values means statistical significance.

It can be seen in Table 3. The SA of the peak knee extension angle and peak internal rotation angle (*p* = 0.000; ES = −0.57) was observed a significantly increase by 17% and 47% after fatigued (*p* = 0.008; ES = −0.25) respectively, while the SA of the peak flexion angle was decreased by 5% (*p* = 0.026; ES = 0.19). In comparison, the SA of peak external rotation angle was very significantly smaller (56%) than before fatigue (*p* = 0.002; ES = 0.54). The SA peak abductive moment of bilateral knee joints was observed an increase by 10% (*p* = 0.048; ES = −0.36). After fatigue, SA of knee stiffness on the coronal plane were significantly higher (13%) than those before fatigue (*p* = 0.005; ES = −0.41).

**TABLE 3:** Changes of knee joint angle, joint moment, range of motion and joint stiffness symmetry before and after fatigue.

Knee	Symmetry angle (%)			
	Pre- mean (±SD)	Post- mean (±SD)	Sig.	ES (Cohen’s d)
Joint angle				
Ext (+)	0.08 (0.07)	0.25 (0.26)	0.008**	−0.25 (−0.53)
Flex (−)	0.09 (0.07)	0.04 (0.03)	0.026*	0.42 (0.93)
Add (+)	0.38 (0.46)	0.36 (0.33)	0.851	0.02 (0.05)
Abd (−)	0.50 (0.41)	0.47 (0.38)	0.773	0.04 (0.08)
Intr (+)	0.17 (0.18)	0.64 (0.45)	0.000**	−0.57 (−1.38)
Extr (−)	0.84 (0.53)	0.28 (0.33)	0.002**	0.54 (1.27)
Joint Moment				
Extn (+)	0.18 (0.13)	0.25 (0.26)	0.327	−0.17 (−0.34)
Flex (−)	0.16 (0.12)	0.18 (0.16)	0.630	−0.07 (−0.14)
Add (+)	0.23 (0.14)	0.27 (0.11)	0.276	−0.15 (−0.32)
Abd (−)	0.26 (0.13)	0.36 (0.13)	0.048*	−0.36 (−0.77)
Intr (+)	0.24 (0.13)	0.29 (0.17)	0.311	−0.16 (−0.33)
Extr (−)	0.23 (0.16)	0.26 (0.21)	0.569	−0.08 (−0.16)
Joint stiffness				
Ext (+)/Flex (−)	0.16 (0.13)	0.21 (0.17)	0.388	−0.16 (−0.33)
Add (+)/Abd (−)	0.19 (0.13)	0.32 (0.16)	0.005**	−0.41 (−0.89)
Intr (+)/Extr (−)	0.23 (0.16)	0.28 (0.16)	0.288	−0.15 (−0.31)

*means significance (*p* < 0.05), “**” means very significance (*p* < 0.01).

Left: Left lower limb (non-dominant). Right: Right (dominant) lower limb. Ext: Extension, Flex: Flexion, Add: Adduction, Abd: Abduction. Intr: Internal rotation, Extr: External rotation. ES: effect Size.

Bold values means statistical significance.

It can be seen form Table 4 that the SA of hip joint of peak flexion angle (*p* = 0.002; ES = −0.54), adduction angle (*p* = 0.001; ES = −0.57), and internal rotation angle (*p* = 0.000; ES = −0.66) significantly increased by 12%, 39% and 36% respectively after fatigue. However, the SA of the peak abduction (*p* = 0.004; ES = 0.39) and the external rotation angle (*p* = 0.000; ES = 0.07) was observed a significantly decrease (39% and 47% respectively). The SA of the peak flexion moment was significantly increased by 11% after fatigue than before (*p* = 0.001; ES = −0.50). The SA of stiffness in the coronal plane were significantly higher (9%) after fatigue than before (*p* = 0.001; ES = −0.50).

**TABLE 4:** Changes of hip joint angle, joint moment, range of motion and joint stiffness symmetry before and after fatigue.

Hip	Symmetry angle (%)			
	Pre- mean (±SD)	Post- mean (±SD)	Sig.	ES (Cohen’s d)
Joint angle				
Flex (+)	0.03 (0.02)	0.15 (0.13)	0.002**	−0.54 (−1.29)
Ext (−)	0.37 (0.35)	0.16 (0.29)	0.082	0.31 (0.65)
Add (+)	0.11 (0.09)	0.50 (0.39)	0.001**	−0.57 (−1.38)
Abd (−)	0.78 (0.52)	0.39 (0.40)	0.004**	0.39 (0.84)
Intr (+)	0.13 (0.08)	0.49 (0.28)	0.000**	−0.66 (−1.75)
Extr (−)	0.84 (4.47)	0.37 (0.43)	0.000**	0.07 (0.15)
Joint Moment				
Flex (+)	0.04 (0.03)	0.15 (0.13)	0.001**	−0.50 (−1.17)
Ext (−)	0.10 (0.09)	0.08 (0.08)	0.226	0.12 (0.23)
Add (+)	0.14 (0.10)	0.15 (0.09)	0.927	−0.05 (−0.11)
Abd (−)	0.17 (0.11)	0.24 (0.11)	0.147	−0.30 (−0.64)
Intr (+)	0.20 (0.12)	0.27 (0.14)	0.100	−0.26 (−0.54)
Extr (−)	0.23 (0.13)	0.22 (0.15)	0.929	0.04 (0.07)
Joint stiffness				
Flex (+)/Ext (−)	0.07 (0.07)	0.08 (0.07)	0.798	−0.07 (−0.14)
Add (+)/Abd (−)	0.13 (0.09)	0.22 (0.16)	0.031*	−0.33 (0.69)
Intr (+)/Extr (−)	0.18 (0.12)	0.24 (0.14)	0.256	−0.22 (−0.46)

*means significance (*p* < 0.05), “**” means very significance (*p* < 0.01).

Left: Left lower limb (non-dominant). Right: Right (dominant) lower limb. Flex: Flexion, Ext: Extension, Add: Adduction, Abd: Abduction. Intr: Internal rotation, Extr: External rotation. ES: effect Size.

Bold values means statistical significance.

## Discussion

The current study showed that biomechanical parameters of the right (dominant) and the left (non-dominant) lower limbs were asymmetric before and after fatigue, and the SA of some parameters changed after fatigue, which was consistent with hypotheses 1) and 2). Interestingly, the symmetry of some parameters weakened after fatigue, and symmetry of a few kinematic parameters increased after fatigue, which was not consistent with hypothesis 3) in this study.

Congenital slight asymmetry exists in the anthropometry and function of the human body (Kujanová et al., 2008). The differences observed in the pre-fatigue stage may be related to the biomechanics and neuromuscular asymmetry of the body (Sadeghi et al., 2000). More internal rotation of hip rotation may be caused by weaker external rotation muscles (Powers, 2010). Therefore, the further analysis of EMG (Electromyography) signals of bilateral external rotation muscles may explain the phenomenon that the left hip joint showed a large internal rotation angle in current study. Likewise, the foot axis angle reflects the degree of the foot in and out of rotation in the horizontal plane, excessive external rotation of the left ankle after fatigue was found in this study, which may be related to the result that the non-dominant limb is mainly engaged in stabilization work (Yan et al., 2013). The runners in this study presented the left ankle with more significant adduction moment and joint stiffness, suggesting that the lateral load of the plantar can be transferred to relatively stable and keep good joint stability, provide better support in the gait period stabilize posture, to the next gait cycle and demonstrates advantages in advance of the prepared limbs (Sadeghi et al., 2000). Significant abduction moment of the right ankle may cause the short-term collapse of the medial longitudinal arch (Mei et al., 2019) to ensure sufficient propulsion of dominant planter during the gait support (Sadeghi et al., 2000). Joint stiffness is considered the critical factor in stabilizing the limb movement itself without interference and the greater joint stiffness can prevent joint instability (Brughelli and Cronin, 2008). The greater joint stiffness in the sagittal plane and coronal plane in the left knee joint was observed in this study suggesting that the non-dominant limb contribute more stability during gait stance phase. However, Lambach et al. found no significant difference adduction moment of bilateral knee joints in a study of walking gait (Lathrop-Lambach et al., 2014), such inconsistencies may be caused by differences gait speed. In addition, a study investigating running gait showed that a high-impact activity compared with walking required increased muscle activity to attenuate impact (Echeverria et al., 2010). The more significant adduction moment in the left knee presented in this study may be associated with the risk of knee osteoarthritis (Chang et al., 2010), which may be proves that non-dominant limbs often have more risk of injury than dominant limbs (Hayes et al., 2004).

Brown et al. (Brown et al., 2014) observed that the symmetry of the lower limbs did not change after running fatigued and the neuromuscular fatigue rates of both limbs were the same, the reason the current study contradicts this conclusion may be due to gender differences of participants. In addition, the symmetry of the knee joint parameters deteriorated most obviously after fatigue, and the symmetry of kinematics and kinetics parameters decreases in all three anatomic aspects, suggesting that the fatigue variation rate of bilateral limbs is different in running gait. This deterioration of asymmetry may be associated with the risk of overuse injury of the knee joint, which may be caused by a high susceptibility to fatigue in the knee motor muscles of the non-dominant limb (Gao et al., 2020a). Moreover, the previous study reported that the hip and knee muscles work together to control various degrees of hip freedom during the exhausted running (Hayes et al., 2004). The asymmetry of hip flexion angle and moment significantly increased, suggesting that the asymmetry of the lower extremities may change from distal to proximal as fatigue develops. Furthermore, the adduction angle and joint stiffness of hip flexion also become more asymmetrical, which may be due to insufficient central nervous system motor neuron drive and reduced bilateral symmetry due to peripheral changes in hip muscle level during fatigue (Gandevia, 2001; He and Fekete, 2021). Therefore, the biomechanical variability of the bilateral limbs caused by fatigue is not the same, and the risk of injury related to body control maybe increase after fatigue (Winter et al., 2016; Dempster et al., 2021; Xiang et al., 2022; Xu et al., 2022). However, part of kinematics parameters, according to the current study found no symmetry enhanced after fatigue phenomenon. Ankle and hip SA abduction angle decreased, indicating that the two joints abduction symmetry after fatigue is rather strong, and it can be interpreted as the body response in order to maintain the overall stability of the lower limbs after the fatigue of a compensation mechanism (Gao et al., 2020a). Moreover, the more symmetry of flexion angle of the knee in this study may be a positive adjustment of the neuromuscular system to avoid overuse injury after fatigue (Vagenas and Hoshizaki, 1991). Another exciting result of current study is that all three anatomic joints become more asymmetrical in terms of internal rotation at the horizontal plane. In contrast, all joints become more symmetrical in terms of external rotation. This phenomenon may be a coordination mechanism generated by the body to maintain the symmetry and stability of the overall lower limbs after fatigue (Heil et al., 2020).

This study explored the biomechanical differences and symmetry changes of the bilateral lower limbs of amateur runners before and after running-induced fatigue experiments. However, there are still three limitations. First of all, the running-induced fatigue experiment in this study was carried out on a treadmill. In contrast, the biomechanical test before and after fatigue was carried out on the ground, ignoring the possible experimental errors caused by different planes. Secondly, the symmetry evaluation adopts extreme values, and a discrete data set, while ignoring data continuity. Future studies should consider using other methods, such as SI_Nigg_ (Nigg et al., 2013), to evaluate the symmetry of time series data. In addition, the results of this study are valid for young male amateur only and in female runners and elite runners the data should be confirmed with further studies.

## Conclusion

Overall, this study reveals the effect of running fatigue on the symmetry of joint angles, moment, and stiffness of amateur runners’ lower limbs. The results shown that the lower limbs of amateur male runners were asymmetrical in both pre- and post-fatigued states during running. In addition, running fatigue resulted in an increased asymmetry of load on the hip, knee and ankle joints of the lower extremities. Future research is needed to investigate the relationship between fatigue-induced joints load asymmetry and running-related injuries development. The external rotation angle of three joints become more symmetry after fatigued, suggesting that fatigue dose not deteriorate the symmetry of all biomechanical parameters especially the joint angles in the horizontal plane. The knowledge of the effects of fatigue on lower extremity biomechanics may have implications for the training of long-distance running footwork, such as musculature development, to prevent load accumulation in unilateral joints and improve the efficiency of long-distance running.

## Data Availability

The original contributions presented in the study are included in the article/supplementary material, further inquiries can be directed to the corresponding authors.

## References

[B1] Abd-Elfattah H. M. Abdelazeim F. H. Elshennawy S. (2015). Physical and cognitive consequences of fatigue: A review. J. Adv. Res. 6, 351–358. 10.1016/j.jare.2015.01.011 26257932 PMC4522584

[B2] Barber S. D. Noyes F. R. Mangine R. E. Hartman W. (1990). Quantitative assessment of functional limitations in normal and anterior cruciate ligament-deficient knees. Clin. Orthop. Relat. Res. 255, 204–214. 10.1097/00003086-199006000-00028 2347154

[B3] Beck O. N. Azua E. N. Grabowski A. M. (2018). Step time asymmetry increases metabolic energy expenditure during running. Eur. J. Appl. Physiol. 118, 2147–2154. 10.1007/s00421-018-3939-3 30027520

[B4] Bell D. R. Pennuto A. P. Trigsted S. M. (2016). The effect of exertion and sex on vertical ground reaction force variables and landing mechanics. J. Strength Cond. Res. 30, 1661–1669. 10.1519/JSC.0000000000001253 26562710

[B43] Binkley J. M. Stratford P. W. Lott S. A. Riddle D. L. (1999). The lower extremity functional scale (LEFS): scale development, measurement properties, and clinical application. Phys Ther. 79, 371–383. 10.1093/ptj/79.4.371 10201543

[B5] Brown A. M. Zifchock R. A. Hillstrom H. J. (2014). The effects of limb dominance and fatigue on running biomechanics. Gait Posture 39, 915–919. 10.1016/j.gaitpost.2013.12.007 24405748

[B6] Brughelli M. Cronin J. (2008). Influence of running velocity on vertical, leg and joint stiffness : Modelling and recommendations for future research. Sports Med. 38, 647–657. 10.2165/00007256-200838080-00003 18620465

[B7] Butler R. J. Crowell H. P. Iii Davis I. M. (2003). Lower extremity stiffness: Implications for performance and injury. Clin. Biomech. 18, 511–517. 10.1016/S0268-0033(03)00071-8 12828900

[B8] Cen X. Lu Z. Baker J. S. István B. Gu Y. (2021). A comparative biomechanical analysis during planned and unplanned gait termination in individuals with different arch stiffnesses. Appl. Sci. 11, 1871. 10.3390/app11041871

[B9] Ceyssens L. Vanelderen R. Barton C. Malliaras P. Dingenen B. (2019). Biomechanical risk factors associated with running-related injuries: A systematic review. Sports Med. 49, 1095–1115. 10.1007/s40279-019-01110-z 31028658

[B10] Chakravarty E. F. Hubert H. B. Lingala V. B. Fries J. F. (2008). Reduced disability and mortality among aging runners: A 21-year longitudinal study. Arch. Intern. Med. 168, 1638–1646. 10.1001/archinte.168.15.1638 18695077 PMC3175643

[B11] Chang A. Hochberg M. Song J. Dunlop D. Chmiel J. S. Nevitt M. (2010). Frequency of varus and valgus thrust and factors associated with thrust presence in persons with or at higher risk of developing knee osteoarthritis. Arthritis Rheum. 62, 1403–1411. 10.1002/art.27377 20213800 PMC2921866

[B12] Dempster J. Dutheil F. Ugbolue U. C. (2021). The prevalence of lower extremity injuries in running and associated risk factors: A systematic review. Phys. Activity Health 5 (1), 133–145. 10.5334/paah.109

[B13] Dierks T. A. Davis I. S. Hamill J. (2010). The effects of running in an exerted state on lower extremity kinematics and joint timing. J. Biomech. 43, 2993–2998. 10.1016/j.jbiomech.2010.07.001 20663506

[B14] Echeverria J. C. Rodriguez E. Velasco A. Alvarez-Ramirez J. (2010). Limb dominance changes in walking evolution explored by asymmetric correlations in gait dynamics. Phys. A Stat. Mech. its Appl. 389, 1625–1634. 10.1016/j.physa.2009.12.025

[B15] Furlong L.-A. Egginton N. L. (2018). Kinetic asymmetry during running at preferred and non-preferred speeds. Med. Sci. Sports Exerc. 50, 1241. 10.1249/MSS.0000000000001560 29360663

[B16] Gandevia S. C. (2001). Spinal and supraspinal factors in human muscle fatigue. Physiol. Rev. 81, 1725–1789. 10.1152/physrev.2001.81.4.1725 11581501

[B17] Gao Z. Mei Q. Fekete G. Baker J. S. Gu Y. (2020a). The effect of prolonged running on the symmetry of biomechanical variables of the lower limb joints. Symmetry 12, 720–731. 10.3390/sym12050720

[B18] Gao Z. Mei Q. Xiang L. Gu Y. (2020b). Difference of walking plantar loadings in experienced and novice long-distance runners. Acta Bioeng. Biomech. 22, 127–147. 10.37190/ABB-01627-2020-02 33518724

[B19] Hamill J. Moses M. Seay J. (2009). Lower extremity joint stiffness in runners with low back pain. Res. Sports Med. 17, 260–273. 10.1080/15438620903352057 19967604

[B20] Hannigan J. Pollard C. D. (2020). Differences in running biomechanics between a maximal, traditional, and minimal running shoe. J. Sci. Med. Sport 23, 15–19. 10.1016/j.jsams.2019.08.008 31501022

[B21] Hayes P. R. Bowen S. J. Davies E. J. (2004). The relationships between local muscular endurance and kinematic changes during a run to exhaustion at v VO2max. J. Strength Cond. Res. 18, 898–903. 10.1519/r-13503.1 15586950

[B22] He Y. Fekete G. (2021). The effect of cryotherapy on balance recovery at different moments after lower extremity muscle fatigue. Phys. Activity Health 5, 255–270. 10.5334/paah.154

[B23] Heil J. Loffing F. Büsch D. (2020). The influence of exercise-induced fatigue on inter-limb asymmetries: A systematic review. Sports Med. Open 6, 39–16. 10.1186/s40798-020-00270-x 32844254 PMC7447715

[B24] Hulme A. Nielsen R. O. Timpka T. Verhagen E. Finch C. (2017). Risk and protective factors for middle-and long-distance running-related injury. Sports Med. 47, 869–886. 10.1007/s40279-016-0636-4 27785775

[B25] Koblbauer I. F. Van Schooten K. S. Verhagen E. A. Van Dieën J. H. (2014). Kinematic changes during running-induced fatigue and relations with core endurance in novice runners. J. Sci. Med. Sport 17, 419–424. 10.1016/j.jsams.2013.05.013 23790535

[B26] Kujanová M. Bigoni L. Velemínská J. Velemínský P. (2008). Limb bones asymmetry and stress in medieval and recent populations of Central Europe. Int. J. Osteoarchaeol. 18, 476–491. 10.1002/oa.958

[B27] Lathrop-Lambach R. L. Asay J. L. Jamison S. T. Pan X. Schmitt L. C. Blazek K. (2014). Evidence for joint moment asymmetry in healthy populations during gait. Gait Posture 40, 526–531. 10.1016/j.gaitpost.2014.06.010 25035185 PMC4267535

[B28] Lee D.-C. Pate R. R. Lavie C. J. Sui X. Church T. S. Blair S. N. (2014). Leisure-time running reduces all-cause and cardiovascular mortality risk. J. Am. Coll. Cardiol. 64, 472–481. 10.1016/j.jacc.2014.04.058 25082581 PMC4131752

[B29] Mei Q. Gu Y. Xiang L. Baker J. S. Fernandez J. (2019). Foot pronation contributes to altered lower extremity loading after long distance running. Front. Physiol. 10, 573. 10.3389/fphys.2019.00573 31191329 PMC6540596

[B30] Nigg S. Vienneau J. Maurer C. Nigg B. M. (2013). Development of a symmetry index using discrete variables. Gait Posture 38, 115–119. 10.1016/j.gaitpost.2012.10.024 23218726

[B31] Powers C. M. (2010). The influence of abnormal hip mechanics on knee injury: A biomechanical perspective. J. Orthop. Sports Phys. Ther. 40, 42–51. 10.2519/jospt.2010.3337 20118526

[B32] Sadeghi H. Allard P. Prince F. Labelle H. (2000). Symmetry and limb dominance in able-bodied gait: A review. Gait Posture 12, 34–45. 10.1016/S0966-6362(00)00070-9 10996295

[B33] Seeley M. K. Umberger B. R. Shapiro R. (2008). A test of the functional asymmetry hypothesis in walking. Gait Posture 28, 24–28. 10.1016/j.gaitpost.2007.09.006 17997095

[B34] Smeets A. Vanrenterghem J. Staes F. Verschueren S. (2019). Match play induced changes in landing biomechanics with special focus on fatigability. Med. Sci. Sports Exerc. 51, 1884–1894. 10.1249/MSS.0000000000001998 30933003

[B35] Vagenas G. Hoshizaki B. (1991). Functional asymmetries and lateral dominance in the lower limbs of distance runners. J. Appl. Biomechanics 7, 311–329. 10.1123/ijsb.7.4.311

[B36] Van Gent R. Siem D. Van Middelkoop M. Van Os A. Bierma-Zeinstra S. Koes B. (2007). Incidence and determinants of lower extremity running injuries in long distance runners: A systematic review. Br. J. Sports Med. 41, 469–480. 10.1136/bjsm.2006.033548 17473005 PMC2465455

[B37] Winter S. Gordon S. Watt K. (2016). Effects of fatigue on kinematics and kinetics during overground running: A systematic review. J. Sports Med. Phys. Fit. 57, 887–899. 10.23736/s0022-4707.16.06339-8 27074435

[B38] Wouters I. Almonroeder T. Dejarlais B. Laack A. Willson J. D. Kernozek T. W. (2012). Effects of a movement training program on hip and knee joint frontal plane running mechanics. Int. J. Sports Phys. Ther. 7, 637–646. 23316427 PMC3537459

[B39] Xiang L. Mei Q. Wang A. Shim V. Fernandez J. Gu Y. (2022). Evaluating function in the hallux valgus foot following a 12-week minimalist footwear intervention: A pilot computational analysis. J. Biomech. 132, 110941. 10.1016/j.jbiomech.2022.110941 35063832

[B40] Xu D. Quan W. Zhou H. Sun D. Baker J. S. Gu Y. (2022). Explaining the differences of gait patterns between high and low-mileage runners with machine learning. Sci. Rep. 12 (1), 2981. 10.1038/s41598-022-07054-1 35194121 PMC8863837

[B41] Yan S.-H. Zhang K. Tan G.-Q. Yang J. Liu Z.-C. (2013). Effects of obesity on dynamic plantar pressure distribution in Chinese prepubescent children during walking. Gait Posture 37, 37–42. 10.1016/j.gaitpost.2012.05.018 22858245

[B42] Zifchock R. A. Davis I. Higginson J. Royer T. (2008). The symmetry angle: A novel, robust method of quantifying asymmetry. Gait Posture 27, 622–627. 10.1016/j.gaitpost.2007.08.006 17913499

